# Synaptic depression in the CA1 region of freely behaving mice is highly dependent on afferent stimulation parameters

**DOI:** 10.3389/fnint.2013.00001

**Published:** 2013-01-25

**Authors:** Jinzhong J. Goh, Denise Manahan-Vaughan

**Affiliations:** ^1^Department of Neurophysiology, Medical Faculty, Ruhr University BochumBochum, Germany; ^2^International Graduate School of NeuroscienceBochum, Germany

**Keywords:** *in vivo*, murine, hippocampus, LTD, STD

## Abstract

Persistent synaptic plasticity has been subjected to intense study in the decades since it was first described. Occurring in the form of long-term potentiation (LTP) and long-term depression (LTD), it shares many cellular and molecular properties with hippocampus-dependent forms of persistent memory. Recent reports of both LTP and LTD occurring endogenously under specific learning conditions provide further support that these forms of synaptic plasticity may comprise the cellular correlates of memory. Most studies of synaptic plasticity are performed using *in vitro* or *in vivo* preparations where patterned electrical stimulation of afferent fibers is implemented to induce changes in synaptic strength. This strategy has proven very effective in inducing LTP, even under *in vivo* conditions. LTD *in vivo* has proven more elusive: although LTD occurs endogenously under specific learning conditions in both rats and mice, its induction has not been successfully demonstrated with afferent electrical stimulation alone. In this study we screened a large spectrum of protocols that are known to induce LTD either in hippocampal slices or in the intact rat hippocampus, to clarify if LTD can be induced by sole afferent stimulation in the mouse CA1 region *in vivo*. Low frequency stimulation at 1, 2, 3, 5, 7, or 10 Hz given in the range of 100 through 1800 pulses produced, at best, short-term depression (STD) that lasted for up to 60 min. Varying the administration pattern of the stimuli (e.g., 900 pulses given twice at 5 min intervals), or changing the stimulation intensity did not improve the persistency of synaptic depression. LTD that lasts for at least 24 h occurs under learning conditions in mice. We conclude that a coincidence of factors, such as afferent activity together with neuromodulatory inputs, play a decisive role in the enablement of LTD under more naturalistic (e.g., learning) conditions.

## Introduction

Synaptic plasticity describes the ability of neurons to modify the strength or efficacy of their signal transmission across synapses. Patterned stimulation of afferent fibers to the hippocampus can induce persistent changes in synaptic transmission that is capable of lasting for hours or days and even up to weeks (Bliss and Lomo, 1973; Abraham et al., 1994; Abraham, 2003). These changes can comprise enhancements of synaptic efficacy, known as long-term potentiation (LTP) or reductions in efficacy, known as long-term depression (LTD). New, relevant information is believed to be stored through long-lasting changes in synaptic strength across the synapses in the neural circuits involved in the encoding process. This process can be mimicked by electrical stimulation of afferent fibers to the hippocampus, whereby LTP is typically elicited via application of a short and brief stimulation (e.g., 0.5 s, 1 s) at high frequencies (e.g., 50 Hz, 100 Hz), and is often dependent on the activation of the ionotropic N-methyl-D-aspartate (NMDA) glutamate receptors (Bliss and Collingridge, 1993; Huang et al., 1996). Repetition of the trains of stimulation further enhances the amplitude and persistency of the synaptic potentiation (Frey et al., 1993; Huang and Kandel, 1994; Huang et al., 1994). LTD, on the other hand, can be electrically induced through low-frequency stimulation typically in the range of 1–5 Hz given as single pulses (Brandon et al., 1995; Otani and Connor, 1996; Manahan-Vaughan and Reymann, 1997; Oliet et al., 1997; Nicoll et al., 1998; Manahan-Vaughan et al., 1999; Manahan-Vaughan, 2000a; Mockett et al., 2002; Cao et al., 2004; Kemp and Manahan-Vaughan, 2004; Massey et al., 2004; Xiong et al., 2004; Jo et al., 2006; Neyman and Manahan-Vaughan, 2008; Seoane et al., 2009; Popkirov and Manahan-Vaughan, 2011) but can also be elicited by afferent stimulation with pairs of stimuli in quick succession (i.e., 50 ms) at low frequencies (Kemp and Bashir, 1997, 1999; Huber et al., 2000; Kemp et al., 2000; Moult et al., 2008; Caruana et al., 2011). The activation of the NMDA receptors, like in LTP, is known to be crucial for the induction of LTD in the hippocampus CA1 region (Dudek and Bear, 1992; Heynen et al., 1996).

Although the discovery of LTD succeeded that of LTP, this form of synaptic plasticity has become increasingly interesting due to accumulating evidence as to its prominence in learning and memory functions, especially in the various hippocampal sub-regions (Kemp and Manahan-Vaughan, 2004, 2007, 2008b; Etkin et al., 2006; Hagena and Manahan-Vaughan, 2011; Goh and Manahan-Vaughan, 2012a). Most of the studies conducted on LTD in the intact animal have been done in rats. Here, LTD that lasts for days and weeks has been described in this species (Abraham et al., 1994; Heynen et al., 1996; Manahan-Vaughan and Braunewell, 1999). Although a multitude of studies have examined LTD in the murine hippocampal slice preparation, very little data exists as to the conditions under which LTD is expressed in the intact mouse. Furthermore, studies in murine slices typically follow LTD for periods of maximally 1 h after induction, thus studies as to the longevity of LTD in the mouse hippocampus, and the underlying mechanisms are largely missing. This comprises a serious knowledge deficit, as so many conclusions about the mechanisms underlying persistent LTD are made on the basis of short-term studies conducted in slice preparations.

In this study, we characterized the susceptibility of the Schaffer collateral–CA1 synapses to express synaptic depression in response to patterned afferent electrical stimulation in the intact, freely behaving mouse. A broad spectrum of afferent stimulation parameters were tested that are known to induce synaptic depression in rats *in vitro* (Otani and Connor, 1996; Kemp and Bashir, 1997; Oliet et al., 1997; Nicoll et al., 1998; Kemp and Bashir, 1999; Huber et al., 2000; Kemp et al., 2000; Mockett et al., 2002; Massey et al., 2004; Bartlett et al., 2007; Moult et al., 2008; Caruana et al., 2011) and *in vivo* (Manahan-Vaughan and Reymann, 1997; Manahan-Vaughan et al., 1999; Manahan-Vaughan, 2000a; Cao et al., 2004; Kemp and Manahan-Vaughan, 2004; Xiong et al., 2004; Neyman and Manahan-Vaughan, 2008; Popkirov and Manahan-Vaughan, 2011), as well as in mice *in vitro* (Brandon et al., 1995; Etkin et al., 2006; Michaelsen et al., 2010). The ability of monosynaptic transmission across the Schaffer collateral–CA1 synapses to express depression in response to electrical stimulation was characterized mainly according to stimulation frequency, number and pattern but also stimulation intensity. Our goal was to clarify if, and to what extent hippocampal LTD is expressed in the freely behaving mouse following patterned electrical stimulation of afferent fibers.

## Materials and methods

### Laboratory animals

The present study was carried out in accordance with the European Communities Council Directive of September 22nd, 2010 (2010/63/EU) for care of laboratory animals and after approval of the local ethics committee (Bezirksamt Arnsberg). All efforts were made to minimize animal suffering and to reduce the number of animals. Male C57/BL6 mice (7–8 weeks at the time of surgery; acquired from Charles River, Germany) were used in all of the experiments. All mice attained the minimum weight of 22 g before being subjected to surgical electrode implantation. The mice were housed in individual cages in a temperature- (22 ± 2°C) and humidity- (55 ± 5%) controlled vivarium (Scantainer Ventilated Cabinets, Scanbur A/S, Denmark) with a constant 12-h light–dark cycle (lights on from 8 a.m. to 8 p.m.) where they had access to food and water *ad libitum*. Prior to surgery, the animals were housed in groups of maximally six animals in a single cage. All surgical procedures and experiments were conducted during the day. The mice were anesthetized (sodium pentobarbital 60 mg/kg, i.p.) and underwent stereotaxic chronic implantation of bipolar stimulating electrodes in the right Schaffer collateral pathway of the dorsal hippocampus [anterioposterior (AP): −2.0 mm; mediolateral (ML): 2.0 mm from bregma; dorsoventral (DV): ~1.4 mm from brain surface] and monopolar recording electrodes in the right ipsilateral CA1 *stratum radiatum* (AP: −1.9; ML: 1.4; DV: ~1.2) to monitor the evoked potentials at the Schaffer Collateral–CA1 synapses. The stimulating and recording electrodes were made of polyurethane-coated stainless steel wire (100 μm diameter; Gündel, BioMedical Intruments, Germany) and were lowered into the brain through a hole drilled on the skull. On the contralateral side, two holes were drilled on the skull into which anchor screws were inserted. The anchor screws were attached to stainless steel wires (A-M Systems, USA) which served as reference and ground electrodes. The five wires were secured on a six-pin socket (Conrad Electronic SE, Germany) and the whole assembly was stabilized on the skull using dental cement. Test-pulse recordings during surgery aided depth adjustment of the electrodes, which was later verified by postmortem histology. After surgery, mice were housed individually and given at least 7 days recovery time before experiments began. Electrophysiological recordings and behavioral paradigms were performed in 20 (L) × 20 (W) × 30 (H) cm topless recording chambers wherein the mice could move freely and had access to food and water *ad libitum*. Animals were transferred in their cages into the experiment room 1 day before the start of experiments to ensure adequate acclimatization to the gross environment.

### Measurement of evoked potentials

Each mouse had its socket connected via a swivel connector to the recording/stimulation system wires suspended above the recording chamber to enable monitoring of evoked potentials while the animal freely behaved and performed tasks. The field excitatory post-synaptic potential (fEPSP) was employed as a measure of excitatory synaptic transmission in the CA1 region. To obtain these measurements, an evoked response was generated in the *stratum radiatum* by stimulating the Schaffer collateral at low frequency (0.025 Hz) with single biphasic square waves of 0.2 ms duration per half-wave, generated by a constant current isolation unit (World Precision Instruments, USA). The fEPSP signal was amplified using a differential AC amplifier (A-M Systems, USA) and digitalized through a data acquisition unit (Cambridge Electronic Design, UK). For each time point measured during the experiments, five consecutively evoked fEPSP responses at 40 s intervals were averaged. The first six time-points, which were recorded at 5 min intervals, were averaged and all time points were expressed as a mean percentage (± standard error of the mean) of this value. Plasticity-inducing electrical stimulation, together with the relevant behavioral task (when appropriate) was applied immediately after the sixth time point and synaptic transmission was recorded for another 4 h (240 min). A further 1 h recording was performed the next day, roughly 24 h after the experiment began to determine the degree of persistency of any changes in synaptic transmission. fEPSP was quantified by measuring the slope obtained on the first negative deflection of the evoked potential. By means of an input-output curve determination conducted before every experiment, the largest obtainable fEPSP was found for each individual animal (maximum intensity used 125 μA). The intensity that elicited 40% of the maximum fEPSP was used for recordings. Electroencephalography (EEG) activity was monitored throughout the course of the experiment for the occurrence of seizure activity. No behavioral signs or EEG activity indicating seizures were observed.

### Electrical induction of synaptic plasticity

All animals were first tested in a “baseline” experiment without any external behavioral stimuli to ensure that the recordings were stable. The following protocols were used to examine the frequency-dependency of synaptic depression in the intact mouse hippocampus: 1 Hz (900 stimuli), 3 Hz (200 stimuli), 3 Hz (300 stimuli), 3 Hz (900 stimuli), 3 Hz (1200 stimuli), 3 Hz (3 trains of 300 stimuli each with 5 min inter-train intervals), 3 Hz (2 trains of 900 stimuli each with 5 min inter-train intervals), 5 Hz (300 stimuli), 7 Hz (100 stimuli), 7 Hz (300 stimuli), 10 Hz (100 stimuli), and 10 Hz (300 stimuli) (Table 1). All aforementioned stimulation protocols, used to examine synaptic depression, were applied at a stimulation intensity that produces 70% of the largest inducible fEPSP response, as determined in the I/O curve. Sixty percent intensity was used in one experiment (3 Hz 900 stimuli) to examine the role of stimulation intensity on synaptic depression.

**Table 1:** **Summary of stimulation parameters and evoked responses 5 min, 30 min, 1 h, and 4 h post-stimulation**.

**Stimulation parameters**		**5 min**	**30 min**	**1 h**	**4 h**
No stimulation (control)		101.2	101.0	100.1	97.4
1 Hz	900 pulses	104.5	99.6	102.0	101.5
	450 paired pulses	101.2	101.9	99.0	97.7
2 Hz	300 pulses	101.7	101.6	105.2	100.8
	1800 pulses	110.3	122.7	118.6	117.9
3 Hz	200 pulses	28.5	93.9	94.9	93.9
	300 pulses	12.5	77.9	104.2	96.0
	900 pulses	58.6	77.8	90.1	113.4
	900 pulses 60%	113.0	106.5	104.4	103.2
	300 pulses 3 trains	79.1	94.5	100.0	110.6
	900 pulses 2 trains	81.1	102.4	107.8	113.3
	1200 pulses	109.8	123.4	132.3	124.3
5 Hz	300 pulses	50.9	93.2	104.0	111.3
	900 pulses	78.2	91.2	97.0	109.1
7 Hz	100 pulses	70.1	100.7	100.6	93.9
	300 pulses	14.63	95.1	102.7	109.6
10 Hz	100 pulses	22.5	92.7	98.3	96.2
	300 pulses	71.0	104.0	102.5	105.3

*The table describes CA1 responses to patterned afferent stimulation at 1, 3, 5, 7, or 10 Hz or to an equivalent control period where no stimulation was given (“No stimulation”). The pulse number is also shown. Where different stimulus intensities were used these are listed [e.g., 60% of the maximal field excitatory post-synaptic potential (fEPSP) response obtained in the input-output (i/o) curve]. In all other cases, the simulation intensity used was 70% of the maximal i/o response. In some cases (listed) stimulation was given as 2 or 3 trains 5 min apart. Otherwise stimulation was always given in single train of pulses. Mean fEPSP values (represented as % baseline) are shown for the time-points comprising 5 min, 30 min, 1 h, or 4 h after application of patterned stimulation*.

### Histological analysis

**Figure 1:**
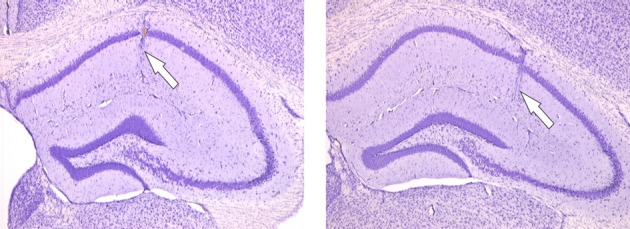
**Nissl-stained microphotographs showing the hippocampus electrode positions.** Photographs of hippocampal slices following the Nissl staining procedure. The white arrows point approximately to the final position of the recording electrode in the CA1 *stratum radiatum* dendritic layer on the left photograph and to the final position of the stimulating electrode in the Schaffer collaterals on the right photograph respectively.

Postmortem histological analysis of the electrode localization from the surgical implantation was performed to verify whether the electrodes were positioned in their respective desired positions. The entire brain of an animal was first carefully removed from the cranium. Special attention was paid to avoid exertion of physical pressure onto the brain that could result in the damage of cortical structures and confound histological analyses. Upon removal, the brains were transferred to a small vial and the tissue was immediately fixed in 4% paraformaldehyde (PFA; IUPAC name polyoxymethylene) solution in phosphate buffered saline (PBS) at a pH of 7.4 for 2 h. The tissue was then dehydrated in a stepwise fashion by immersing it in progressively increasing concentrations of glycerol (10 and 20%). This ensured that water was appropriately removed from the brain to prevent the formation of water crystals within the tissue during the freezing procedure later that could cause tissue damage. After dehydration, the brain was shock-frozen in isopentane and stored at −70°C. The frozen tissue was cut coronally on a microtome into slices of 50 μm thickness. The slices were stored in 0.1 ml PBS and mounted in a 45% sodium chloride solution onto 4% potassium chrome alum-gelatin coated glass slides. The mounted slices were left to air-dry for 7 days on the glass slides. When dried, the glass slides were placed for 45 min in a 1:1 mixture of chloroform and 96% ethanol and washed in distilled water. The slices were then stained in 0.5% cresyl violet and the staining was further differentiated with 50% ethanol and acetate. The slices were dehydrated again in ascending concentrations of alcohol and finally degreased in xylene. Embedding was carried out with DePex mounting medium for histology (Serva Electrophoresis GmbH, Germany) and images were taken a digital image acquisition camera (Model Kappa DX30, Kappa Optronics GmbH, Germany) (Figure 1).

### Data analysis

The results across animals were expressed in terms of mean ± s.e.m. To analyze the electrophysiological data between groups, a analysis of variance (ANOVA) followed by *post-hoc* Fisher LSD test was used. The fEPSPs from the period after electrical stimulation to the end of the experiment was compared. The significance level was set at *p* < 0.05.

## Results

### Synaptic depression in freely behaving mice is not induced using well-established protocols for rats or *in vitro* experiments

**Figure 2:**
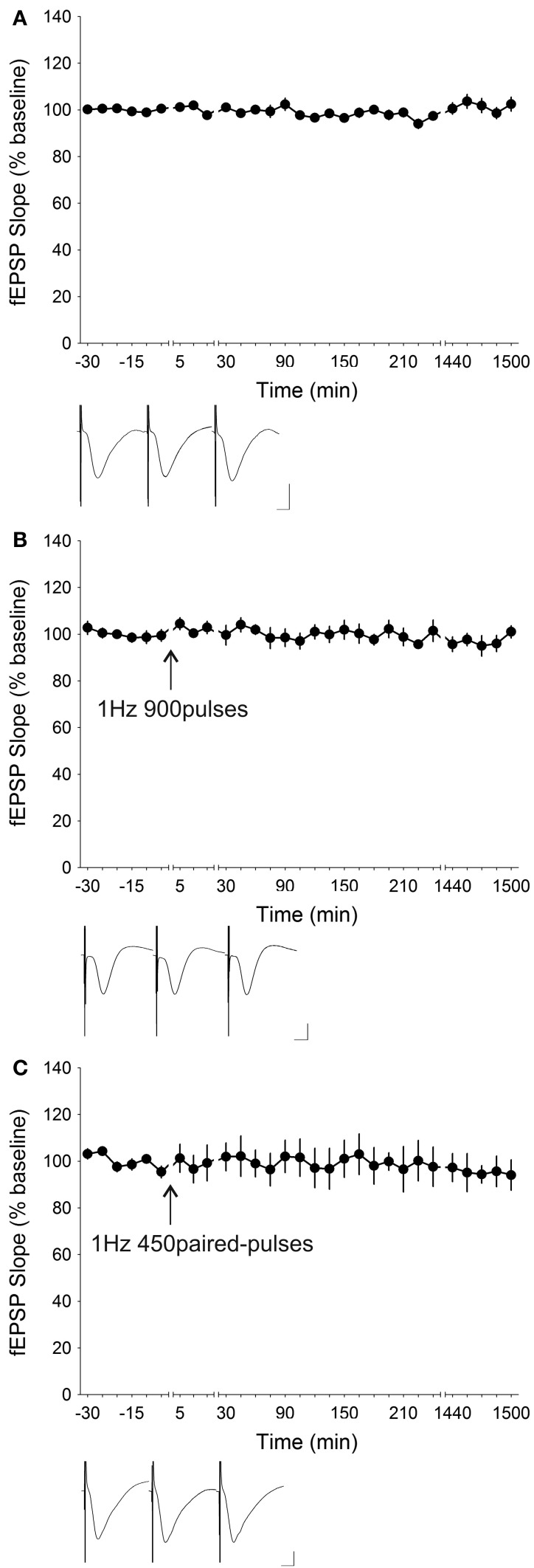
**Low-frequency stimulation at 1 Hz did not induce synaptic depression at the mouse Schaffer collateral–CA1 synapses *in vivo*. (A)** When no external patterned stimulation was applied, the mice (*n* = 18) exhibited stable synaptic transmission in response to test-pulse stimulation (0.025 Hz) throughout the entire course of the recording (1500 min). **(B)** Low-frequency stimulation (LFS) at 1 Hz, 900 stimuli, a stimulation protocol used to induce robust long-term depression (LTD) in freely behaving rats and mouse hippocampal slices, failed to induce any form of detectable synaptic plasticity (ANOVA, *p* = 0.95011) at the CA1 synapses in freely behaving mice (*n* = 7). **(C)** Another commonly applied LFS protocol which consists of 450 pairs of stimuli given at 1 Hz was also incapable of inducing any significantly detectable changes (ANOVA, *p* = 0.07712) in synaptic efficacy in freely behaving mice (*n* = 6). Insets: analog traces illustrating the Schaffer collateral–CA1 field potentials at pre-stimulation, 5 min and 24 h (1440 min). Vertical scale bar corresponds to 2 mV and horizontal scale bar corresponds to 5 ms.

LFS given at 1 Hz (900 stimuli) is known to induce robust LTD that lasts for at least 24 h in freely behaving rats (Manahan-Vaughan and Reymann, 1997; Manahan-Vaughan et al., 1999; Kemp and Manahan-Vaughan, 2004; Neyman and Manahan-Vaughan, 2008; Popkirov and Manahan-Vaughan, 2011). This LFS protocol has also been proven to be effective in eliciting LTD in rats and mice *in vitro* (Brandon et al., 1995; Kemp et al., 2000; Massey et al., 2004; Etkin et al., 2006; Bartlett et al., 2007; Michaelsen et al., 2010). Therefore, we first tested whether this particularly salient LTD-inducing protocol was capable of producing similar effects in the freely behaving mouse *in vivo*. Surprisingly, in contrast to rats and *in vitro* mouse experiments, 1 Hz LFS (900 stimuli) was ineffective in inducing any form of synaptic plasticity in freely behaving mice (*n* = 7) (Figure 2B). The resulting synaptic profile was not statistically different from basal values without LFS application [ANOVA, *F*_(1, 23)_ = 0.0040, *p* = 0.95011]. The results of the various stimulation protocols used were statistically compared to basal synaptic transmission without the application of any patterned afferent electrical stimulation (*n* = 18), which was stable across a 24 h recoding period (Figure 2A).

**Figure 3:**
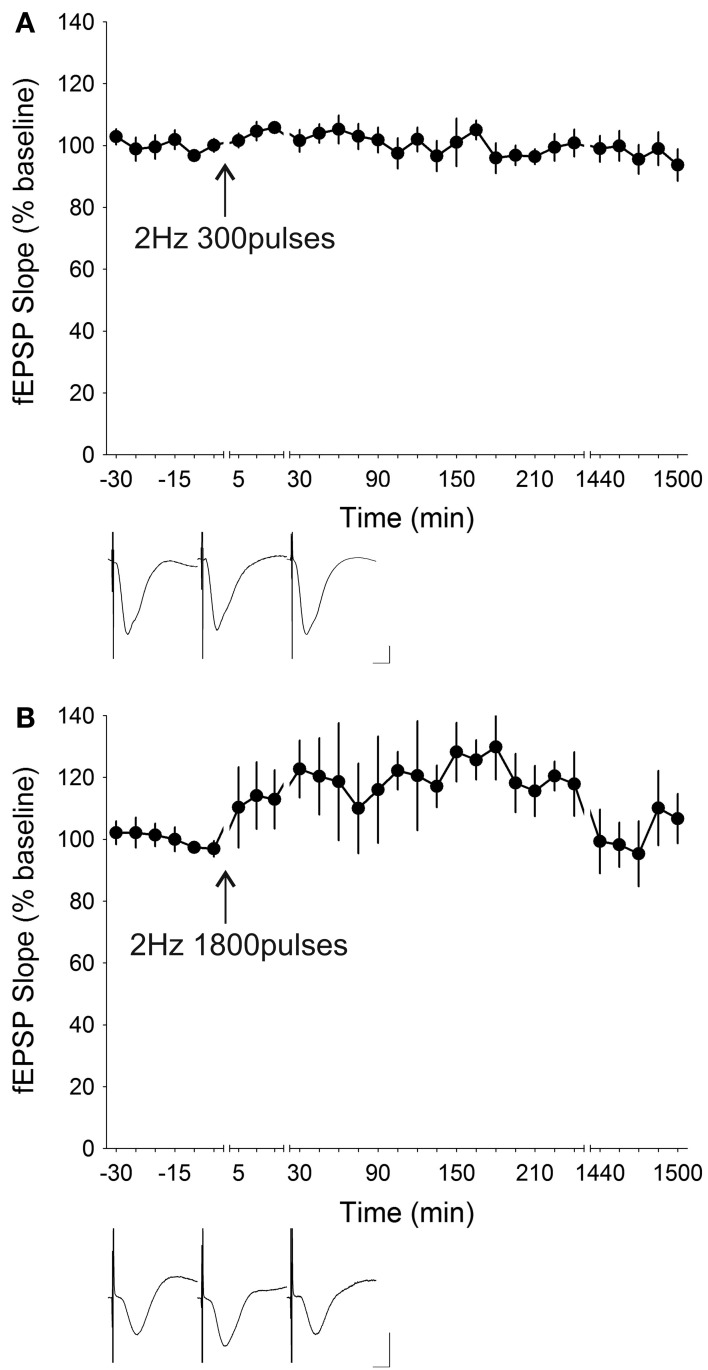
**Low-frequency stimulation at 2 Hz did not induce synaptic depression but initiated slow-onset potentiation at higher number of stimuli. (A)** Low-frequency stimulation (LFS) applied at 2 Hz for 300 pulses did not result in a significant change (ANOVA, *p* = 0.42910) in synaptic efficacy at the CA1 synapses in freely behaving mice (*n* = 8). **(B)** Increasing the number of stimuli to 1800 at 2 Hz led to a significant slow-onset potentiation (ANOVA, *p* < 0.0001; *n* = 6). Insets: analog traces illustrating the Schaffer collateral–CA1 field potentials at pre-stimulation, 5 min and 24 h (1440 min). Vertical scale bar corresponds to 2 mV and horizontal scale bar corresponds to 5 ms.

LFS comprising 450 pairs of stimuli (900 stimuli in total) given at 1 Hz with 50 ms between the pairs of stimuli has been shown to be robustly effective in inducing LTD *in vitro* (Kemp and Bashir, 1997, 1999; Huber et al., 2000; Kemp et al., 2000; Moult et al., 2008; Caruana et al., 2011). Application of this particular protocol to freely behaving mice (*n* = 6) did not result in a response that significantly deviated [ANOVA, *F*_(1, 19)_ = 3.4932, *p* = 0.07712] from the baseline transmission profile (Figure 2C). Increasing the frequency to 2 Hz, whilst reducing the number of stimuli to 300, also failed to elicit any form of depression [ANOVA, *F*_(1, 20)_ = 0.65143, *p* = 0.42910; *n* = 8] (Figure 3A). This again was in contrast to the ability of several different rat strains to express short-term depression (STD) in response to 2 Hz LFS stimulation *in vivo* (Manahan-Vaughan, 2000a), as well as *in vitro* (Otani and Connor, 1996). On the other hand, raising the number of stimuli to 1800 times induced a slow-onset potentiation that was statistically significant [ANOVA, *F*_(1, 21)_ = 23.625, *p* < 0.0001; *n* = 6] (Figure 3B).

### Synaptic depression induction in freely behaving mice is highly specific for frequency, stimulus intensity, number of stimuli, and pattern of stimulation

**Figure 4:**
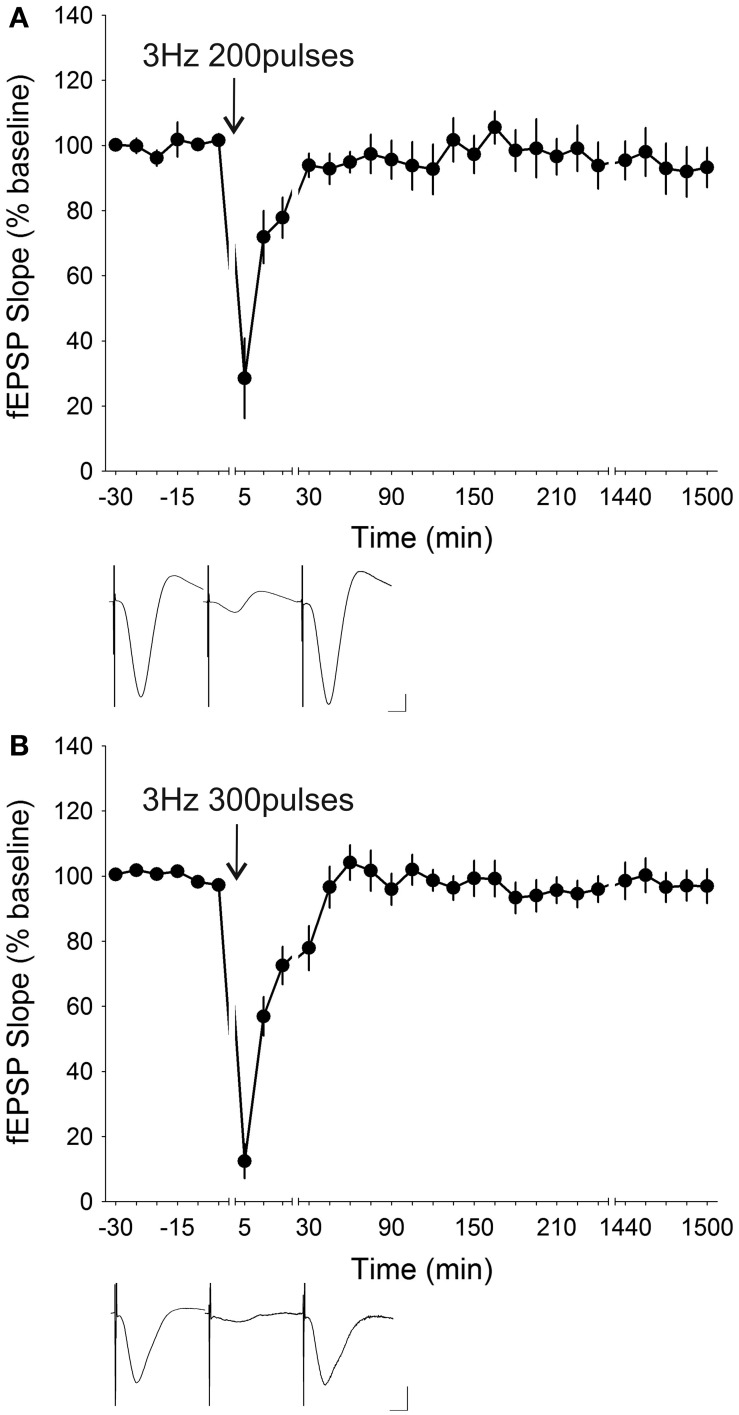
**Low-frequency stimulation at 3 Hz given for 200 or 300 times induced robust short-term depression. (A)** Low-frequency stimulation (LFS) given at 3 Hz for 200 stimuli (ANOVA, *p* < 0.05; *n* = 6) and **(B)** 300 stimuli (ANOVA, *p* < 0.05; *n* = 9) both induced robust and significant short-term depression (STD). Increasing the number of stimuli by 100 from 200 to 300 at 3 Hz resulted in an enhancement in the initial synaptic depression from 28.52 ± 12.28% to 12.46 ± 5.35% whilst *post-hoc* analysis revealed an increase in the duration of significant synaptic depression from 15 min to 30 min post-stimulation. Insets: analog traces illustrating the Schaffer collateral–CA1 field potentials at pre-stimulation, 5 min and 24 h (1440 min). Vertical scale bar corresponds to 2 mV and horizontal scale bar corresponds to 5 ms.

**Figure 5:**
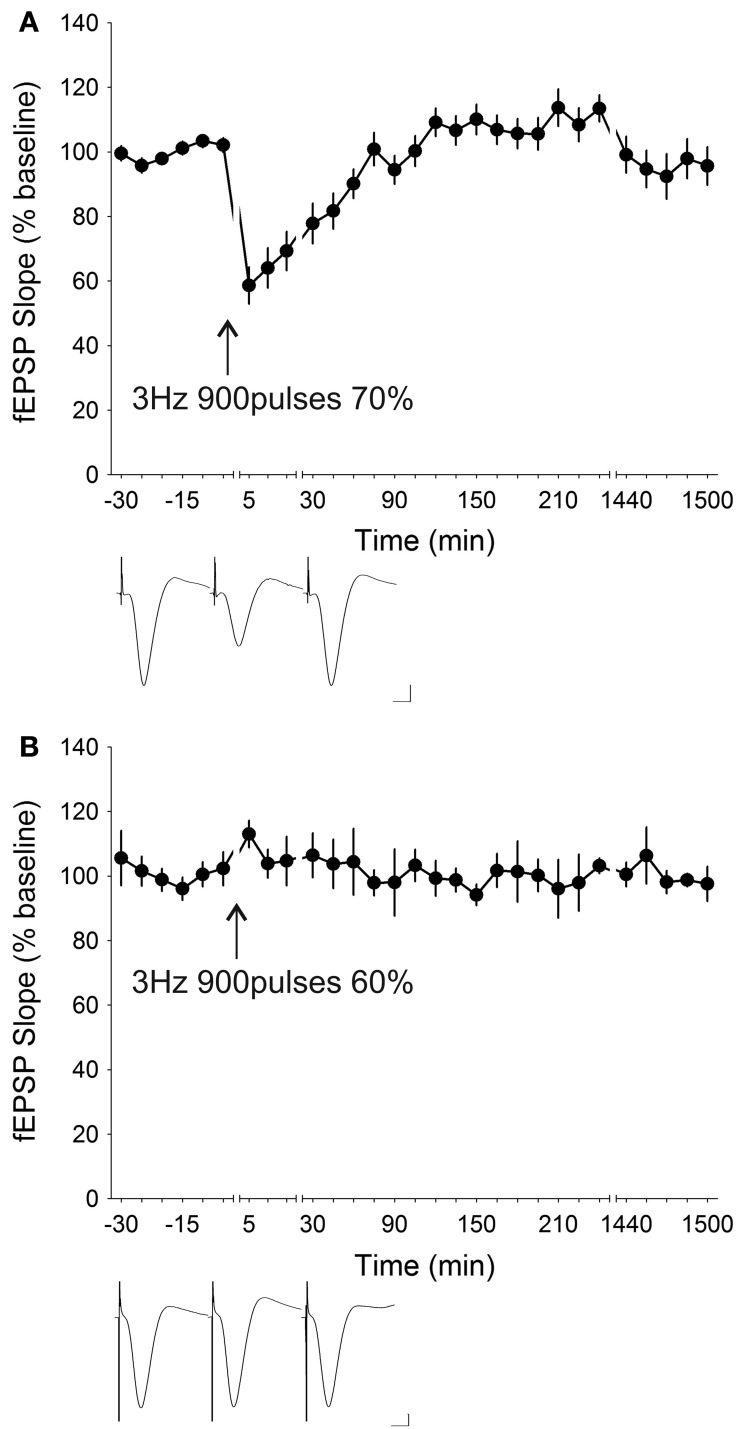
**The extent of short-term depression induced by low-frequency stimulation at 3 Hz depends on the number of stimuli and the stimulation intensity. (A)** Further increasing the number of stimuli to 900 times at 3 Hz resulted in an STD that was depressed (ANOVA, *p* = 0.16118; *n* = 14) for 60 min post-stimulation. At 900 stimuli initial synaptic depression was, however, considerably ameliorated (58.64 ± 5.74%) compared to at 200 and 300 stimuli and a late-onset potentiation developed after approximately 60 min. **(B)** Decreasing the stimulation intensity used for 3 Hz 900 stimuli LFS to 60% of the maximum inducible fEPSP which was aimed at circumventing the late-onset synaptic potentiation failed to induce any significant (ANOVA, *p* = 0.70365; *n* = 6) form of synaptic plasticity at the CA1 synapses. Insets: analog traces illustrating the Schaffer collateral–CA1 field potentials at pre-stimulation, 5 min and 24 h (1440 min). Vertical scale bar corresponds to 2 mV and horizontal scale bar corresponds to 5 ms.

LFS at 3 Hz is known to induce synaptic depression to differing extents in both freely behaving rats (Manahan-Vaughan, 2000a; Cao et al., 2004; Xiong et al., 2004) as well as in hippocampal slices (Mockett et al., 2002). When applied for 200 pulses in freely behaving mice (*n* = 6), 3 Hz LFS evoked significant [ANOVA, *F*_(1, 23)_ = 5.8358, *p* < 0.05] STD which lasted for approximately 30 min before return to baseline levels (Figure 4A). The initial depression of the evoked potential after LFS was reduced to 28.52 ± 12.28% of the pre-stimulation level and *post-hoc* analysis revealed that the fEPSP responses were significantly depressed for the first 15 min succeeding stimulation. Increasing the number of stimuli by an additional 100 times to a total of 300 stimuli at 3 Hz induced marked STD [ANOVA, *F*_(1, 25)_ = 6.8927, *p* < 0.05; *n* = 9] of a comparable profile (Figure 4B). Here, the initial depression was reduced to 12.46 ± 5.35% and *post-hoc* test showed that responses were significantly depressed for the first 30 min after LFS. Application of the 3 Hz LFS for 900 stimuli (*n* = 14) resulted in synaptic depression [ANOVA, *F*_(1, 31)_ = 2.0605, *p* = 0.16118] (Figure 5A). A *post-hoc* test showed that the significant initial depression was prolonged by 30–60 min compared to that elicited with 300 stimuli. Despite the longer initial depression, the drawback of administering 900 stimuli was that an overshoot developed. This resulted in a gradual potentiation of the fEPSP responses in the later phase of recordings, which was sporadically marginally significant after 2 h post-LFS. Intended at eliminating the late-phase potentiation, a lowered stimulation strength was used. Rather than using 70% of the maximum inducible fEPSP, 60% was employed as the LFS intensity for the same 3 Hz 900 times protocol (Figure 5B). Interestingly, this reduction in stimulation strength completely abolished synaptic depression resulting in a profile not dissimilar from basal transmission [ANOVA, *F*_(1, 22)_ = 0.14852, *p* = 0.70365; *n* = 6], thus indicating that a stimulus intensity of 60% was insufficient to induce synaptic depression at 3 Hz stimulation.

**Figure 6:**
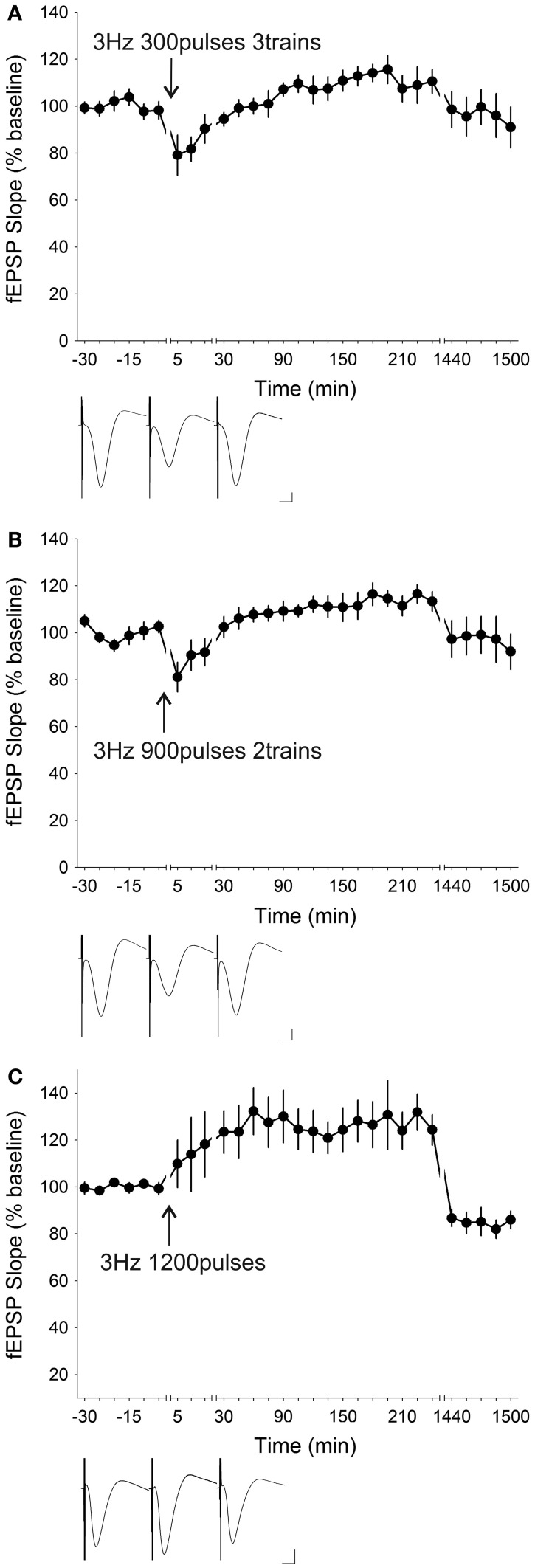
**Varying the pattern of stimulation or increasing the number of stimuli at 3 Hz did not enhance but ameliorated synaptic depression. (A)** Application of 900 stimuli in 3 trains of 300 stimuli (5 min inter-train interval) led to an initial depression of 79.08 ± 8.59% which was significantly depressed for 10 min post-stimulation and a late-onset potentiation that was significant between 150 min to 180 min (ANOVA, *p* = 0.54620; *n* = 6). **(B)** Two trains of 900 stimuli given at 3 Hz induced a synaptic response of similar profile. A small initial synaptic depression (81.12 ± 6.31%) appeared immediately after low-frequency stimulation (LFS) which gradually developed into a significant late-onset potentiation between 90 min to 225 min (ANOVA, *p* < 0.05; *n* = 7). **(C)** LFS given at 3 Hz for 1200 pulses did not induce any synaptic depression but resulted in robust slow-onset potentiation (ANOVA *p* < 0.01; *n* = 6). Insets: analog traces illustrating the Schaffer collateral–CA1 field potentials at pre-stimulation, 5 min and 24 h (1440 min). Vertical scale bar corresponds to 2 mV and horizontal scale bar corresponds to 5 ms.

It was then tested whether giving the stimuli in several trains rather than a single train was capable of enhancing or consolidating the 3 Hz-induced synaptic depression. This protocol was of particular interest since in freely behaving mice it was shown that 100 Hz-induced LTP could be enhanced by given two trains of 50 stimuli rather than a single train of 100 stimuli (Buschler et al., 2012). Application of 3 trains of 300 stimuli (total of 900 stimuli) with an inter-train interval of 5 min at 3 Hz resulted in synaptic profile that tended to be but was not significantly different from baseline [ANOVA, *F*_(1, 22)_ = 0.37569, *p* = 0.54620; *n* = 6] (Figure 6A). The initial depression (79.08 ± 8.59%) was considerably attenuated compared to with one train of 900 pulses (58.64 ± 5.74%). *Post-hoc* test revealed that the depression was significantly reduced up to 10 min post-LFS and the overshoot was significantly potentiated between 150 min and 180 min post-LFS before returning to baseline values. Repeating 900 pulses twice (total of 1800 stimuli) with a 5 min inter-train interval led to a minor initial depression (81.12 ± 6.31%) followed by a significant potentiation [ANOVA, *F*_(1, 22)_ = 4,7481, *p* < 0.05; *n* = 7] centered around 90 min to 225 min before returning to baseline values (Figure 6B). This repetition of 900 stimuli was incapable of enhancing or consolidating the robust STD observed after a single train of 900 pulses. Giving 3 Hz with one continuous train of 1200 pulses did not enhance synaptic depression in any way but resulted in robust synaptic potentiation [ANOVA, *F*_(1, 23)_ = 8,7965, *p* < 0.01; *n* = 6] which was significant after merely 15 min post patterned electrical stimulation and persisted for at approximately 4 h (Figure 6C).

**Figure 7:**
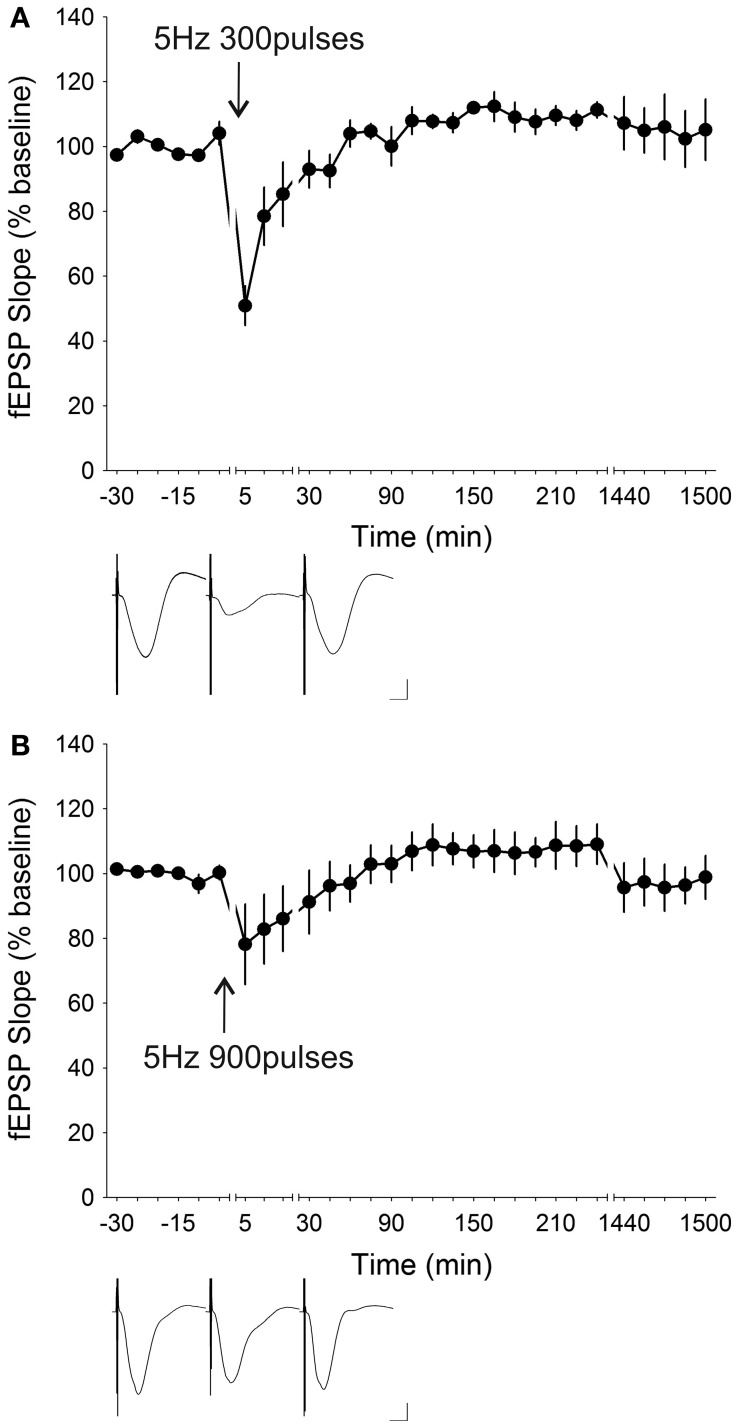
**Stimulation at 5 Hz resulted in varying degrees of synaptic depression depending on stimulation the number of stimuli administered. (A)** Low-frequency stimulation (LFS) at 5 Hz with 300 stimuli induced short-term depression (STD) with an initial synaptic depression of 50.91 ± 6.10% that was significantly depressed for 15 min post-stimulation (ANOVA, *p* = 0.41773; *n* = 5). **(B)** Increasing the number of stimuli at 5 Hz to 900 pulses resulted in an attenuation of the initial synaptic depression (78.17 ± 12.38%) and reduction in the persistence of the synaptic depression to 10 min (ANOVA, *p* = 0.25328; *n* = 7). Insets: analog traces illustrating the Schaffer collateral–CA1 field potentials at pre-stimulation, 5 min and 24 h (1440 min). Vertical scale bar corresponds to 2 mV and horizontal scale bar corresponds to 5 ms.

**Figure 8:**
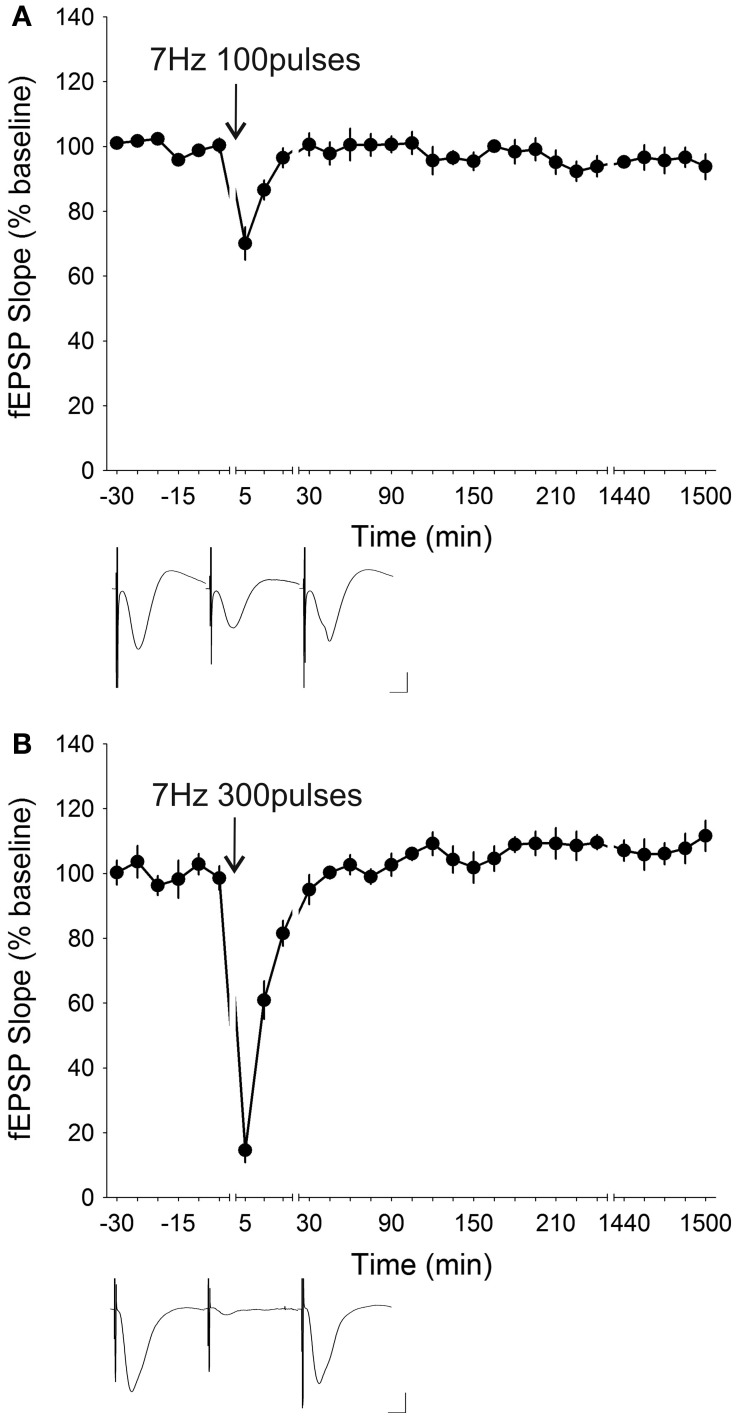
**Stimulation at 7 Hz resulted in varying degrees of synaptic depression depending on the number of stimuli administered. (A)** Low-frequency stimulation (LFS) at 7 Hz elicited a small initial depression of 70.06 ± 5.05% that was significant for up to 10 min post-stimulation when applied for 100 pulses (ANOVA, *p* = 0.27804; *n* = 7). **(B)** Increasing the number of stimuli to 300 pulses at 7 Hz led to an enhancement of the initial depression (14.63 ± 3.81%) as well as the persistence of the depression (ANOVA, *p* = 0.55473; *n* = 8). Insets: analog traces illustrating the Schaffer collateral–CA1 field potentials at pre-stimulation, 5 min and 24 h (1440 min). Vertical scale bar corresponds to 2 mV and horizontal scale bar corresponds to 5 ms.

**Figure 9:**
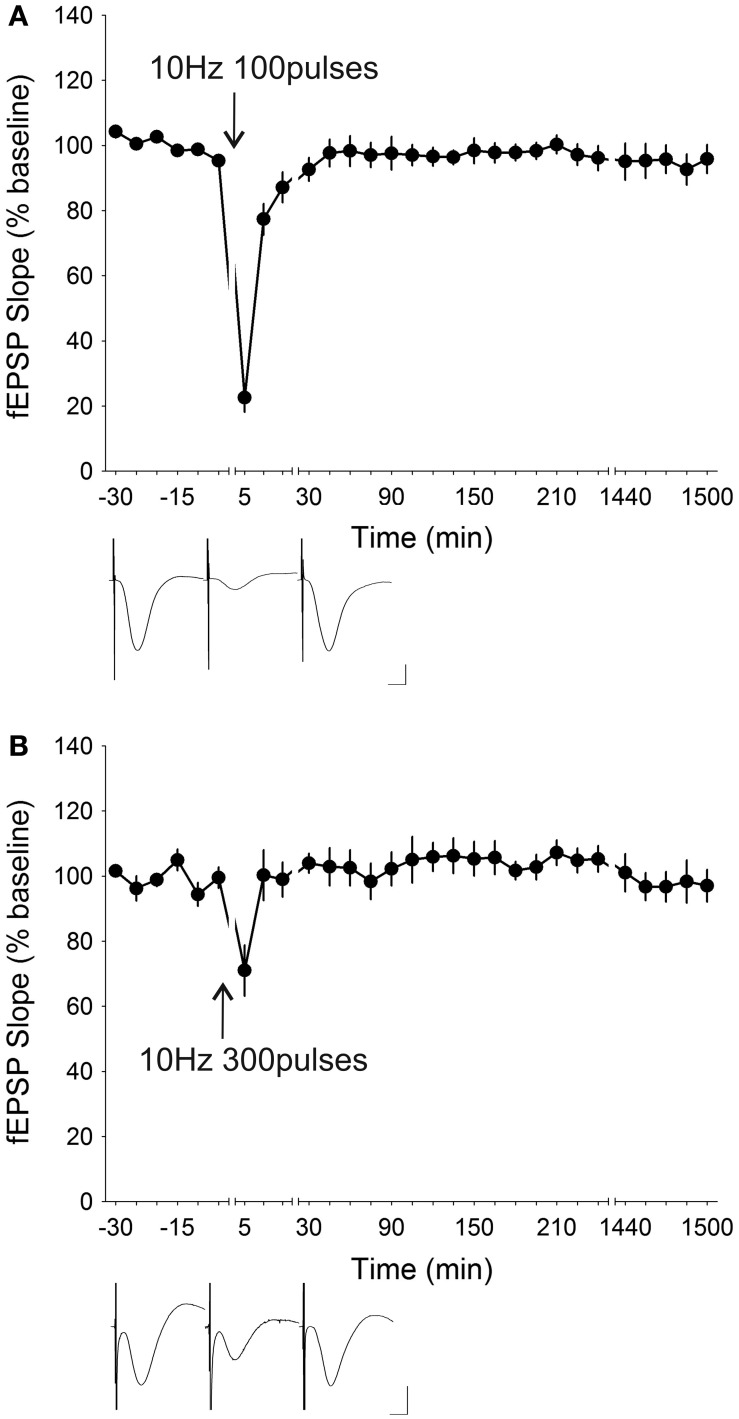
**Stimulation at 10 Hz resulted in varying degrees of synaptic depression depending on the number of stimuli administered. (A)** Low-frequency stimulation (LFS) given at 10 Hz for 100 pulses induced robust STD with an initial synaptic depression of 22.55 ± 4.39% and a significant depression that lasted 15 min post-LFS (ANOVA, *p* < 0.01; *n* = 15). **(B)** Increasing the number of stimuli at 10 Hz to 300 pulses strongly ameliorated the STD resulting in an initial depression of 71.02 ± 7.82% which persisted for 5 min post-stimulation (ANOVA, *p* = 0.71830). Insets: analog traces illustrating the Schaffer collateral–CA1 field potentials at pre-stimulation, 5 min and 24 h (1440 min). Vertical scale bar corresponds to 2 mV and horizontal scale bar corresponds to 5 ms.

LFS at 5 Hz is known to induce synaptic depression *in vitro* in mice (Brandon et al., 1995) and in rats (Oliet et al., 1997; Nicoll et al., 1998) in the hippocampus as well as in other brain regions (Massey et al., 2004; Jo et al., 2006, 2008; Seoane et al., 2009). An attempt to induce synaptic depression in freely behaving mice (*n* = 5) by administering 5 Hz (300 stimuli) resulted in the induction of STD that was only significantly depressed for the first 15 min after stimulation [ANOVA, *F*_(1, 21)_ = 0.68335, *p* = 0.41773] (Figure 7A). When the number of stimuli were raised to 900 pulses, the initial depression was attenuated to 78.17 ± 12.38% from 50.91 ± 6.10% with 300 pulses, and significant synaptic depression lasted for only 10 min post-LFS [ANOVA, *F*_(1, 23)_ = 1.3731, *p* = 0.25328; *n* = 7] (Figure 7B). Increasing the stimulation frequency to 7 Hz whilst reducing the number of stimuli to 100 induced a synaptic depression [ANOVA, *F*_(1, 22)_ = 1.2371, *p* = 0.27804; *n* = 7] which lasted for 10 min post-LFS (Figure 8A). Stimulation at 7 Hz 300 times induced an initial depression that was much more robust (14.63 ± 3.81%) compared to that with 100 stimuli (70.06 ± 5.05%), and a *post-hoc* test showed that the synaptic depression was significant up to 15 min post-LFS [ANOVA, *F*_(1, 24)_ = 0.35890, *p* = 0.55473; *n* = 8] (Figure 8B). A synaptic depression, of similar profile, could be attained by increasing the stimulation frequency to 10 Hz, whilst reducing the number of stimuli to 100 (Figure 9A). Here, the initial depression was robust (22.55 ± 4.39%), and fEPSP responses were significantly depressed for 15 min after stimulation [ANOVA, *F*_(1, 32)_ = 8.8225, *p* < 0.01; *n* = 15]. In contrast, raising the number of stimuli at 10 Hz to 300 pulses resulted in an amelioration of the initial synaptic depression to 71.02 ± 7.82% and a reduction of the duration of synaptic depression that lasted a mere 5 min after LFS [ANOVA, *F*_(1, 23)_ = 0.13337, *p* = 0.71830] without any late-onset potentiation (Figure 9B). The attenuation of the synaptic depression was probably indicative of the wearing down of synaptic machinery as a result of the relatively high stimulation frequency and stimuli number applied.

Thus, across the range of stimulation frequencies and protocols tested, 3, 5, 7, and 10 Hz stimulation were able to induce STD (Table 1). The lowest stimulation frequency capable of inducing synaptic depression was, however, at 3 Hz, with the most effective stimulation protocol being single train of 200 stimuli or a single train of 300 stimuli. These two protocols were especially effective in inducing robust initial synaptic depression that lasted for 15–30 min whilst circumventing the late-onset potentiation observed in other stimulation paradigms, hence making them valuable protocols for inducing STD in freely behaving mice. LTD was not induced with the protocols tested.

## Discussion

The data presented in this study show that synaptic depression elicited by patterned electrical stimulation of afferent fibers to the CA1 sub-region of freely behaving mice is relatively short-lived and occurs in response to a very narrow and specific profile of stimulation frequencies, number of stimuli, as well as stimulus intensity. Only STD, defined as synaptic depression lasting for not more than 1–2 h was observed. Both early-LTD (2–4 h) and late-LTD (>4 h) were not observed across the stimulation protocols that were tested.

In contrast, numerous stimulation protocols have been developed that induce synaptic depression in the rat *in vivo* as well as in hippocampal slices derived from both rats and mice. Synaptic depression ranging from 15 min to hours and even days have been demonstrated in the hippocampus of freely behaving rats using a myriad of afferent stimulation protocols. The most effective strategy is 1 Hz stimulation for 15 min (900 stimuli in total) that results in LTD that lasts for days (Manahan-Vaughan and Reymann, 1997; Manahan-Vaughan et al., 1999; Kemp and Manahan-Vaughan, 2004; Neyman and Manahan-Vaughan, 2008; Manahan-Vaughan and Schwegler, 2011; Popkirov and Manahan-Vaughan, 2011). Stimulation at 2 Hz (Manahan-Vaughan, 2000a), 3 Hz (Manahan-Vaughan, 2000a; Cao et al., 2004; Xiong et al., 2004), or 5 Hz (Manahan-Vaughan, 2000a) elicit synaptic depression that lasts for up to 2 h. Strikingly, none of these protocols elicited persistent LTD in the mouse hippocampus *in vivo*.

Most studies on murine LTD have been done *in vitro*, and although a plethora of reports as to successful LTD exist, typically LTD was only followed for up to 60 min in these studies. Here, LTD following 1 Hz stimulation *in vitro* was reported (Brandon et al., 1995; Kemp et al., 2000; Massey et al., 2004; Etkin et al., 2006; Bartlett et al., 2007; Michaelsen et al., 2010), as was LTD following 2 Hz (Otani and Connor, 1996; Zhang et al., 2008), 3 Hz (Mockett et al., 2002), or 5 Hz afferent stimulation (Brandon et al., 1995; Oliet et al., 1997; Nicoll et al., 1998). Thus, although we would not define it as LTD, because it did not endure for over 2 h (Manahan-Vaughan, 1997), the depression we obtained here under certain conditions at 3 and 5 Hz lasted for up to 45 min and therefore was comparable to that seen in murine *in vitro* studies.

In marked contrast to the relative ease of inducing persistent synaptic depression in hippocampal slices both in rats and mice as well as in the intact rat brain, it was surprising and even counterintuitive in the current work that synaptic depression in the hippocampal CA1 sub-region of the freely behaving mice is short-lived in comparison, and can only be elicited through a restricted repertoire of stimulation protocols. The current experiments showed that although robust initial depression could be induced, the persistency of the depression was short-term at best. Of all stimulation protocols tested, short-term synaptic depression was only induced from afferent stimulation at 3, 5, 7, and 10 Hz with specific stimulation parameters. The difficulty in the induction of synaptic depression in the hippocampus encountered here is however not an isolated incident. Prior experiments from others have indicated that LTD induction in both rats and mice can be problematic and dependent on multiple variables (Doyle et al., 1997; Kemp et al., 2000; Manahan-Vaughan, 2000a,b; Jones et al., 2001; Blaise and Bronzino, 2003; Bliss and Schoepfer, 2004; Liu et al., 2004; Milner et al., 2004; Blaise et al., 2008; Manahan-Vaughan and Schwegler, 2011).

Amongst the limited number of electrophysiological studies recorded from freely behaving mice, most were conducted in the dentate gyrus area of the hippocampal formation and were conducted with respect to persistent synaptic potentiation (Davis et al., 1997; Errington et al., 1997; Jones et al., 2001; McGehee, 2009; Tang and Dani, 2009; Zhang et al., 2010). The current finding that persistent synaptic depression cannot be elicited in CA1 sub-region of the freely behaving mouse is somewhat in contrast to the robust LTD that can be induced *in vivo* in the dentate gyrus of freely moving mice (Koranda et al., 2008). Even more striking in this case is the fact that whilst application of the classical afferent low-frequency electrical stimulation of 900 pulses at 1 Hz produces stark synaptic depression in the dentate gyrus (Koranda et al., 2008), the same protocol fails completely to induce any observable change in synaptic response in the CA1. Although one of the obvious possibilities for the difference in the expression of LTD could be due to the region in question (i.e., CA1 vs. dentate gyrus), the more likely explanation is that in the intact hippocampus regulatory control restricts the range of afferent activities that lead to LTD, and in addition extrahippocampal neuromodulatory control may play an important part. In line with this, we have observed that persistent LTD that lasts for days *in vivo* can be induced in the mouse hippocampus when afferent test-pulse stimulation (which alone causes no change in efficacy of synaptic transmission), or low-frequency stimulation (which alone leads to STD lasting for 30 min), is given concurrently with behavioral tasks that involve novel spatial learning (Goh and Manahan-Vaughan, 2012a,b). This type of persistent change in synaptic strength is strongly regulated by the dopaminergic and noradrenergic systems, at least in rats, where for example the inhibition of dopamine D1/D5 receptors or of beta-adrenergic receptors prevents this kind of learning-facilitated LTD (Lemon and Manahan-Vaughan, 2006; Kemp and Manahan-Vaughan, 2008a; Lemon et al., 2009; Lemon and Manahan-Vaughan, 2012). Furthermore, the coupling of weak afferent stimulation to the CA1 sub-region in freely behaving rats with the activation of the locus coeruleus (Lemon et al., 2009) or pharmacological activation of the D1/D5 dopamine receptors in the CA1 (Lemon and Manahan-Vaughan, 2006) results in robust synaptic depression. In support of this view, blockade of neuromodulatory systems known to be activated during learning has also been shown to consequently impair hippocampal synaptic plasticity (Frey et al., 1990; Straube and Frey, 2003; Straube et al., 2003; Kemp and Manahan-Vaughan, 2005, 2008a; Lemon and Manahan-Vaughan, 2006; Bergado et al., 2007). Whether this is also the case for the mouse hippocampus remains to be seen.

Neuromodulators are known to play a crucial role in steering the expression of various forms of synaptic plasticity that occur during learning in the hippocampus. The noradrenergic system in the brain, stemming primarily from the locus coeruleus (Loy et al., 1980), is known to strongly influence hippocampal synaptic plasticity. Noradrenergic inputs to the entire hippocampus derive from the locus coeruleus, with major innervations terminating in the dentate gyrus. Notwithstanding, beta-adrenergic receptor distribution appears to be relatively homogenous throughout the hippocampus (Crutcher and Davis, 1980; Crutcher et al., 1981). Correspondingly, it was shown that direct stimulation of the locus coeruleus, which mimics endogenous activation, results in an increase of hippocampal CA1 noradrenaline concentrations (Lemon et al., 2009). Noradrenergic activity through locus coeruleus activation enhances the memory encoding, consolidation, as well as retrieval (Devauges and Sara, 1991; Clark et al., 1995, 1999; Ghacibeh et al., 2006). Conversely, antagonism of the noradrenergic receptors in the CA1 impairs the formation of contextual fear and long-term spatial memories (Ji et al., 2003a,b; Kemp and Manahan-Vaughan, 2008a; Lemon et al., 2009). *In vivo*, both hippocampal LTP and LTD can be facilitated through locus coeruleus stimulation (Thomas et al., 1996; Katsuki et al., 1997; Gelinas and Nguyen, 2005). An interesting property of the locus coeruleus neurons is their unique responsiveness to novel incoming sensory stimuli; the neurons fire phasically when novel changes in the environment or information is presented (Sara et al., 1994; Vankov et al., 1995). Thus, the concurrent activation of the locus coeruleus together with a learning event may be crucial in inducing or facilitating the reorganization of activated neuronal ensembles to rapidly encode the information that is new (Bouret and Sara, 2005). In support of its modulatory role of this nature, blockade of the adrenergic receptors in the hippocampus impairs novel empty space-induced LTP at the dentate gyrus (Straube and Frey, 2003; Straube et al., 2003), as well as learning-facilitated LTD in the CA1 in response to novel contextual spatial features (Kemp and Manahan-Vaughan, 2008a). Taken together, the activation of the locus coerulus in response to novel information and the consequent release of noradrenaline in the hippocampus, appear to serve as a means, not necessarily exclusive, for the hippocampal neurons to identify and encode novel changes in the environment through increased the persistency of synaptic plasticity.

Although the dopaminergic system is most well known in positive reinforcement of reward, addiction and self-stimulation (Shankaranarayana Rao et al., 1998; Nestler, 2005; Arias-Carrion and Poppel, 2007), novel incoming information strongly activates burst firing of the ventral tegmental area (VTA) neurons (Steinfels et al., 1983; Ljungberg et al., 1992; Horvitz et al., 1997), which is the major dopaminergic output in the brain (Gasbarri et al., 1997). The VTA sends afferents to the hippocampus (Gasbarri et al., 1997), and the firing of VTA neurons transmits the novelty signal via the D1/D5 receptors located on the hippocampal pyramidal neurons (Ciliax et al., 2000; Khan et al., 2000). The VTA is thought to enable hippocampal novelty detection by selectively augmenting CA3 input to CA1 via the Schaffer collaterals whilst attenuating direct cortical sensory input via the perforant pathway, thereby reinforcing the novel information encoded into a sustained memory trace (Lisman and Otmakhova, 2001). The activation of dopamine receptors is crucial for the expression of both forms of persistent plasticity, LTP and LTD, in the CA1 *in vivo* and *in vitro* (Chen et al., 1996; Otmakhova and Lisman, 1996; Swanson-Park et al., 1999; Lemon and Manahan-Vaughan, 2006; Hansen and Manahan-Vaughan, 2012). Blockade of the receptor results in the failed detection of spatial novelty as well as failure of the associated LTD facilitation in the hippocampal CA1 (Lemon and Manahan-Vaughan, 2006), which not only grants support to the importance of dopamine in novelty detection but also the plausibility that the CA1 serves as a comparator of stored and incoming information. Since both adrenergic and dopaminergic systems are activated and essential during the acquisition of novel information, it is likely that the concerted activation of both receptor types is required for the persistent changes in CA1 plasticity; blocking either receptor component abolishes LTD in the CA1 (Chen et al., 1996; Lemon and Manahan-Vaughan, 2006; Kemp and Manahan-Vaughan, 2008a; Lemon et al., 2009). Whether altering neuromodulation can enable persistent LTD in the mouse hippocampus remains to be explored.

In conclusion, this study shows that in the freely behaving mouse, parameters that are effective in inducing LTD *in vitro* are not effective. Bearing in mind the caveat that most *in vitro* studies did not examine the longevity of LTD (but were restricted to following LTD for up to 1 h thereby confounding a direct comparison), these observations encourage caution in extrapolating the significance of mechanistic studies of murine LTD *in vitro* for long-term synaptic information storage or persistent synaptic modifications using LTD *in vivo*. Furthermore, these data highlight important differences between rat and mouse hippocampal LTD. In rats *in vivo*, LTD of differing durations can be elicited by a wide range of protocols (Manahan-Vaughan, 2000a) and the range of protocols for LTP induction is even wider (Staubli and Lynch, 1987; Kemp and Manahan-Vaughan, 2004, 2008b; Neyman and Manahan-Vaughan, 2008; Hagena and Manahan-Vaughan, 2012). The intriguing finding that the spectrum of stimulation parameters for induction of synaptic depression *in vitro* is highly restricted is complemented by similar findings with regard to murine LTP *in vivo* (Buschler et al., 2012). Rats and mice implement very different strategies to acquire spatial information (Whishaw, 1995; Prusky et al., 2000), which no doubt reflects their adaptation to their naturalistic habitats and their foraging or food-acquisition behavior. Whether the restricted plasticity response range to patterned afferent stimulation reflects a lesser flexibility in learning or information-storage dynamics, or rather reflects a different evolutionary relevance of murine hippocampal plasticity remains to be clarified.

### Conflict of interest statement

The authors declare that the research was conducted in the absence of any commercial or financial relationships that could be construed as a potential conflict of interest.
